# Genetic Analysis of Tryptophan Metabolism Genes in Sporadic Amyotrophic Lateral Sclerosis

**DOI:** 10.3389/fimmu.2021.701550

**Published:** 2021-06-14

**Authors:** Jennifer A. Fifita, Sandrine Chan Moi Fat, Emily P. McCann, Kelly L. Williams, Natalie A. Twine, Denis C. Bauer, Dominic B. Rowe, Roger Pamphlett, Matthew C. Kiernan, Vanessa X. Tan, Ian P. Blair, Gilles J. Guillemin

**Affiliations:** ^1^ Macquarie University Centre for Motor Neuron Disease Research, Department of Biomedical Sciences, Faculty of Medicine, Health and Human Sciences, Macquarie University, Sydney, NSW, Australia; ^2^ Australian e-Health Research Centre, Commonwealth Scientific and Industrial Research Organization, Health & Biosecurity Flagship, Sydney, NSW, Australia; ^3^ Department of Biomedical Sciences, Faculty of Medicine, Health and Human Sciences, Macquarie University, Sydney, NSW, Australia; ^4^ Applied BioSciences, Faculty of Science and Engineering, Macquarie University, Sydney, NSW, Australia; ^5^ Department of Clinical Medicine, Faculty of Medicine, Health and Human Sciences, Macquarie University, Sydney, NSW, Australia; ^6^ Discipline of Pathology, School of Medical Sciences, University of Sydney, Sydney, NSW, Australia; ^7^ Department of Neuropathology, Royal Prince Alfred Hospital, Sydney, NSW, Australia; ^8^ Brain and Mind Centre, University of Sydney, Sydney, NSW, Australia; ^9^ Institute of Clinical Neurosciences, Royal Prince Alfred Hospital, Sydney, NSW, Australia

**Keywords:** sporadic amyotrophic lateral sclerosis (SALS), whole-genome sequence (WGS), tryptophan, kynurenine pathway (KP), serotonin

## Abstract

The essential amino acid tryptophan (TRP) is the initiating metabolite of the kynurenine pathway (KP), which can be upregulated by inflammatory conditions in cells. Neuroinflammation-triggered activation of the KP and excessive production of the KP metabolite quinolinic acid are common features of multiple neurodegenerative diseases, including amyotrophic lateral sclerosis (ALS). In addition to its role in the KP, genes involved in TRP metabolism, including its incorporation into proteins, and synthesis of the neurotransmitter serotonin, have also been genetically and functionally linked to these diseases. ALS is a late onset neurodegenerative disease that is classified as familial or sporadic, depending on the presence or absence of a family history of the disease. Heritability estimates support a genetic basis for all ALS, including the sporadic form of the disease. However, the genetic basis of sporadic ALS (SALS) is complex, with the presence of multiple gene variants acting to increase disease susceptibility and is further complicated by interaction with potential environmental factors. We aimed to determine the genetic contribution of 18 genes involved in TRP metabolism, including protein synthesis, serotonin synthesis and the KP, by interrogating whole-genome sequencing data from 614 Australian sporadic ALS cases. Five genes in the KP (*AFMID, CCBL1, GOT2, KYNU, HAAO*) were found to have either novel protein-altering variants, and/or a burden of rare protein-altering variants in SALS cases compared to controls. Four genes involved in TRP metabolism for protein synthesis (*WARS*) and serotonin synthesis (*TPH1, TPH2, MAOA*) were also found to carry novel variants and/or gene burden. These variants may represent ALS risk factors that act to alter the KP and lead to neuroinflammation. These findings provide further evidence for the role of TRP metabolism, the KP and neuroinflammation in ALS disease pathobiology.

## Introduction

Amyotrophic lateral sclerosis (ALS) is a devastating neurodegenerative disease caused by the loss of upper and lower motor neurons resulting in progressive muscle weakness, wasting, spasticity and eventual paralysis (1). Disease generally occurs between 50 and 60 years of age, and death usually occurs within three to five years from symptom onset, though survival can vary greatly (2). Ten percent of ALS cases are classified as familial, where there is clear evidence of a family history of disease, while the remaining 90% are considered sporadic (SALS), seemingly occurring at random in the population (3).

The genetics of ALS is heterogenous, with over 40 genes and 850 variants now implicated as causal or associated with the disease (3, 4). In European populations, approximately 60% of familial and 10% of SALS cases are attributed to a known causal mutation in these genes (4–6). Additionally, there is strong evidence of a complex genetic contribution to SALS. Studies on the heritability of the disease suggest that 40-60% of SALS risk may be attributed to genetic factors (7–9). A multi-step hypothesis has been described to explain the late onset and sporadic nature of ALS, whereby six ‘steps’ are required for disease onset to occur (10, 11). These steps may include mutations, genetic risk factors, environmental exposures, or other unknown events. Recent genetic analysis identified genes with an increased load, or burden, of rare protein-altering variants in ALS cases. These included *TBK1* and *NEK1*, as well as known ALS genes, *SOD1, TARDBP* and *OPTN* (12). Gene burden complements the multi-step hypothesis for the late onset of ALS, where the presence of genetic alterations may contribute to presentation of disease (10, 11).

**Figure 1:**
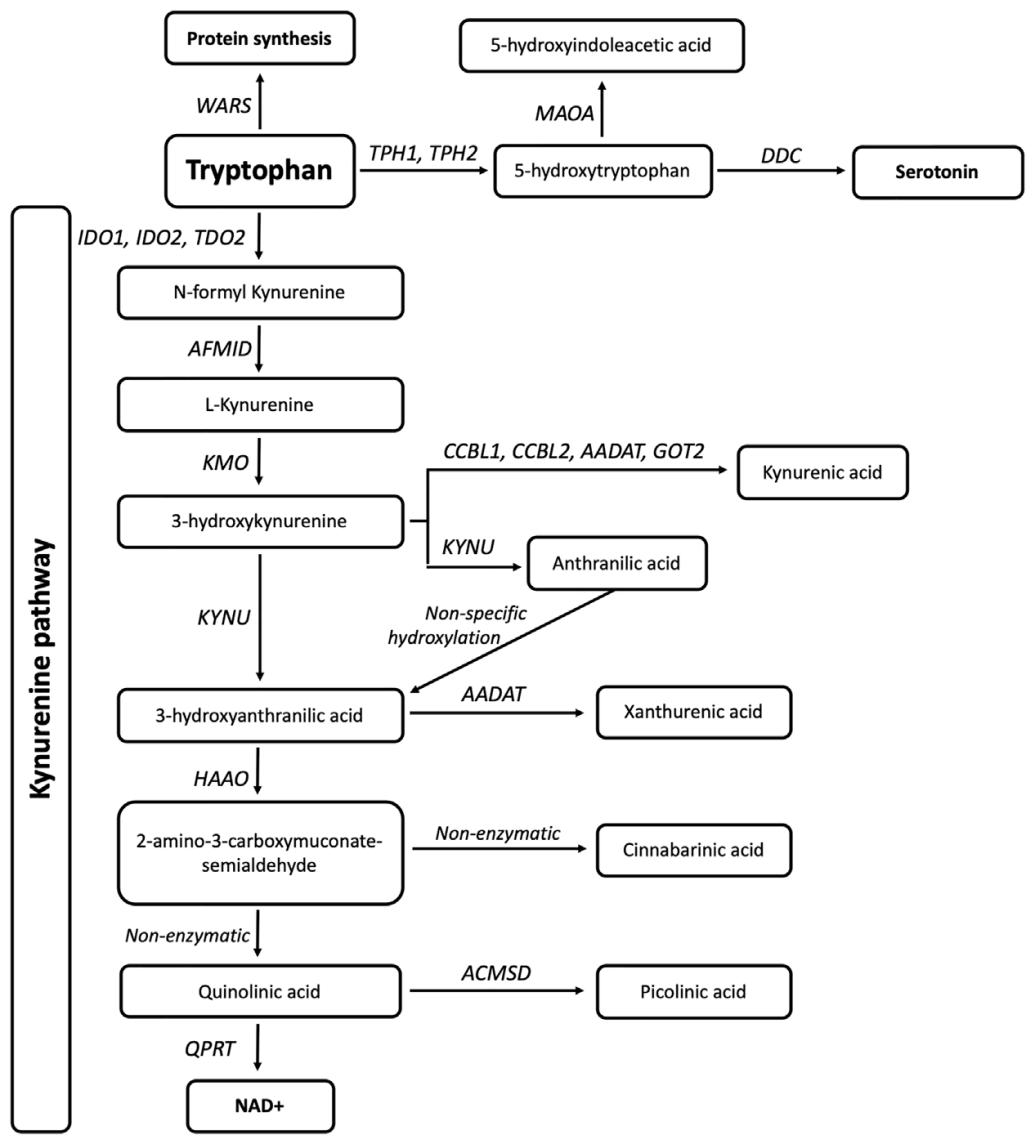
Eighteen genes are involved in tryptophan metabolism and the kynurenine pathway. The gene *WARS* (cytoplasmic tryptophanyl-tRNA synthetase) is responsible for TRP incorporation into proteins, while *TPH1*, *TPH2* (Tryptophan Hydroxylases 1,2), *MAOA* (monoamine oxidase A), and *DDC* (Aromatic L-amino acid decarboxylase/dopa decarboxylase) are involved in serotonin synthesis. *IDO1*, *IDO2* (Indoleamine 2,3-Dioxygenase 1,2) and *TDO2* (Tryptophan 2,3-Dioxygenase) are responsible for the initial and rate limiting step of the KP. This is followed by a molecular cascade to produce active metabolites and ultimately NAD, carried out by *AFMID* (Arylformamidase), *CCBL1*,*CCBL2* (Kynurenine aminotransferase 1,2), *AADAT* (Aminoadipate Aminotransferase), *GOT2* (Glutamic-Oxaloacetic Transaminase 4), *KMO* (Kynurenine 3-Monooxygenase), *KYNU* (Kynureninase), *HAAO* (3-hydroxyanthranilate 3,4-dioxygenase), *ACMSD* (2-amino-3-carboxymuconate-semialdehyde decarboxylase), and *QPRT* (Quinolinate Phosphoribosyltransferase).

Tryptophan (TRP) is an essential amino acid that is either used for the synthesis of proteins, catabolised for the biosynthesis of serotonin and melatonin, or shuttled through the kynurenine pathway (KP) metabolites to produce nicotinamide adenine dinucleotide (NAD^+^). A single enzyme, tryptophanyl-tRNA synthetase, encoded by *WARS* (cytoplasmic) and *WARS2* (mitochondrial), acts in the aminoacylation of TRP to its tRNA for protein synthesis, four enzymes are involved in serotonin synthesis, and 13 enzymes are involved in the KP (**Figure 1**). The KP enzymes act to generate several bioactive intermediates including kynurenine (KYN), kynurenic acid (KYNA), picolinic acid (PIC), quinolinic acid (QUIN) as well as NAD^+^ (13). In physiological conditions, QUIN is usually in low abundance and rapidly transaminated into nicotinic acid, and ultimately NAD^+^. Under neuroinflammatory conditions, QUIN is an excitotoxin that is excessively produced by activated microglia in the brain (14), while KYNA and PIC, produced by astrocytes and neurons respectively, partly prevent QUIN toxicity (14, 15). Increased QUIN levels can amplify neuroinflammation by acting to stimulate neuronal release and inhibit astroglial uptake of glutamate leading to high extracellular glutamate and excitotoxicity, subsequent mitochondrial dysfunction, and activation of proteases (16).

Altered TRP levels and KP dysfunction have been linked to neurodegenerative diseases both genetically and functionally. Multiple mutations in *WARS* have been found to cause distal hereditary motor neuropathy, a form of motor neuron disease characterised by slowly progressive muscle weakness and atrophy (17, 18). Protein-altering missense, nonsense and splicing variants present in KP genes have also been identified as associated with diseases such as multiple sclerosis, Parkinson’s disease, schizophrenia, autism and others (19).

Neuroinflammation and the KP have been functionally implicated in neurodegenerative diseases including ALS (14), multiple sclerosis (20), Parkinson’s (21), Alzheimer’s (22), and Huntington’s Diseases (16). Altered levels of KP metabolites present in cerebrospinal fluid (CSF), serum and spinal cord tissues of ALS patients have been significantly associated with disease. CSF and serum levels of TRP, KYN and QUIN were found to be significantly increased, and serum PIC levels were significantly decreased in ALS patients compared to controls (14). Similarly, KYNA levels in serum was found to be decreased in ALS patients with severe clinical status, as compared to controls. Conversely, in CSF, KYNA levels were lower in controls, indicating a difference in KYNA production between the CNS and blood, as well as the presence of immune activation (23). Additionally, increased levels of IDO1 (the first and rate-limiting enzyme of the KP) and QUIN were identified in the motor cortex and spinal cord of patients (14). KP metabolites (KPMs) also represent promising biomarkers for ALS progression [reviewed in (24)].

Although altered TRP metabolism, serotonin synthesis, the KP and neuroinflammation have all been functionally implicated in ALS, the contribution of variation in key genes from these pathways has not been reported. We aimed to determine the contribution of sequence variants in these genes to ALS through the identification of novel and rare protein-altering variants, and by preforming gene burden analysis in a large cohort of Australian sporadic ALS cases.

## Materials and Methods

### Subjects

Six-hundred and fourteen sporadic ALS cases were recruited through the Macquarie University Neurodegenerative Disease Biobank, Australian MND DNA bank (Royal Prince Alfred Hospital) and the Brain and Mind Centre (University of Sydney). All individuals provided informed consent for research participation as approved by the human research ethics committees of Macquarie University (5201600387), Sydney South West Area Health District and The University of Sydney. All sporadic ALS cases were of predominately European descent, and were diagnosed with probable or definite ALS according to *El Escorial* criteria (25). Demographic characteristics of the cohort, such as sex, age of onset, and mutation status were consistent with that of other European datasets, where a subset of patients carried mutations in known ALS genes including *﻿C9orf72, SOD1* and *TARDBP* or disease associated variation in other ALS genes, as previously reported in McCann et al. (4).

Control genotype data was ascertained from the non-neurological subset of non-Finnish Europeans (nNFE, n=51,592) from the Genome Aggregation Database (gnomAD) (26). Population-specific Australian control genotype data were ascertained through the Diamantina control dataset (AOGC, n=967) and the Medical Genetics Reference Bank (MGRB, n= 1,144) (27). The AOGC dataset comprises of whole-exome sequencing data from neurologically healthy Australians of predominately Western European descent. The MGRB dataset comprises of PCR-amplified whole-genome sequencing data from healthy Australians of >70 years of age and no history of dementia.

### Data Processing

All sporadic ALS samples underwent whole-genome sequencing (WGS, Illumina 150bp PCR-free library, X-Ten sequencer) at The Kinghorn Cancer Centre (Sydney, Australia), as detailed by McCann et al. (4). Data was annotated to hg19 using ANNOVAR and included *in silico* protein prediction tools from the database for non-synonymous SNP’s functional predictions v4.1a (dbNSFP) (4, 28–30). Custom UNIX scripts were used to parse variant call format files for all variants in the target genes. RStudio v3.6.3 (31) was used for all subsequent analyses. Novel variants were considered accurate with base coverage equal or greater than 25X, reference/alternate read depth ratios of 50:50 (^+/-^15%), variant GQ score of 99, and manual IGV visualisation (32).

### Assessment of Genetic Variation

Single nucleotide genetic variation was assessed in the cytoplasmic tryptophanyl-tRNA synthetase gene *WARS* (NM_004184), four genes involved in serotonin metabolism: *DDC*; aromatic L-amino acid decarboxylase/dopa decarboxylase (NM_001082971), *MAOA;* monoamine oxidase A (NM_001270458), *TPH1* and *TPH2;* tryptophan hydroxylases 1,2 (NM_004179 and NM_173353 respectively), and 13 genes involved in the kynurenine pathway: *AADAT;* aminoadipate aminotransferase (NM_016228), *ACMSD;* 2-amino-3-carboxymuconate-semialdehyde decarboxylase (NM_138326), *AFMID;* arylformamidase (NM_001010982), *GOT2;* glutamic-oxaloacetic transaminase 4 (NM_002080), *HAAO;* 3-hydroxyanthranilate 3,4-dioxygenase (NM_012205), *IDO1* and, *IDO2;* indoleamine 2,3-dioxygenase 1,2 (NM_002164 and NM_194294 respectively), *KMO;* kynurenine 3-monooxygenase (NM_003679), *KYAT1/CCBL1* and *KYAT3/CCBL2;* kynurenine aminotransferase 1,2 (NM_001122671 and NM_001008662 respectively), *KYNU;* kynureninase (NM_003937), *QPRT;* quinolinate phosphoribosyltransferase (NM_014298), and *TDO2;* tryptophan 2,3-dioxygenase (NM_005651).

### Variant Filtering and Pathogenicity Scoring

Filtering criteria were applied to identify qualifying variants present in WGS data for burden analysis (both heterozygous and homozygous variants were included). Qualifying variants were defined as those which alter the protein sequence including missense, insertions or deletions, splicing and stop gain or loss variants, and were considered as rare in the population. Rare variants were defined as present at a minor allele frequency (MAF) equal to or less than 0.005, with the exception of the gnomAD nNFE controls, where a MAF equal to or less than 0.0001 was used due the large sample size. Novel genetic variants were defined as those present in SALS, and absent, or only present in a single individual, from all control datasets including the National Centre for Biotechnology Information (NCBI) dbSNP153 database (https://www.ncbi.nlm.nih.gov/snp/).

The potential pathogenicity of novel gene variants was assessed using 12 functional prediction tools from dbNSFP, including SIFT, PolyPhen2-HDIV, PolyPhen2-HVAR, LRT, MutationTaster, MutationAssessor, FATHMM, PROVEAN, MetaSVM, MetaLR, M-CAP and CADD (29). The percentage of deleterious predictions was used to calculate a pathogenicity score, whereby a score of 1 indicates that 100% of tools predicted a deleterious effect. Meta-analysis prediction tools REVEL (nonsynonymous variants only) and BayesDel (nonsynonymous and splicing variants) were also noted from dbNSFP annotation, as these tools were recently found to outperform other *in silico* prediction tools (33–35). Pathogenic cut-off scores were 0.5 for REVEL and -0.057 for BayesDel. The splicing variants were analysed for functional affects using Human Splicing Finder v3.1 (36), NNSplice as part of the MutationTaster tool, CADD and BayesDel. Additional ALS datasets including the ALS Data Browser (ALSdb, New York City, New York (URL: http://alsdb.org) [June 2020]), ALS Variant Server (AVS, Worcester, MA (URL: http://als.umassmed.edu/) [June 2020]), Project MinE (37) [June 2020] and dbGaP (https://www.ncbi.nlm.nih.gov/gap/; Study Accession: phs000101.v5.p1) were also screened for the presence of the novel gene variants identified in Australian SALS cases.

### Gene Burden

Burden analysis was performed on qualifying variants only, as defined above. For burden testing, the total number of qualifying variants per gene in sporadic ALS cases was compared to that of multiple control datasets separately. The Fisher’s exact test (from the R package *exact 2x2*) was used for analysis. As 18 genes were analysed in this project, a Bonferroni correction of the p-value was applied (n=18, p=0.00278).

## Results

Eighteen genes involved in TRP metabolism and the KP (**Figure 1**) were screened for genetic variants in whole-genome sequencing data from 614 Australian sporadic ALS patients. Three-hundred and eleven single nucleotide non-intergenic variants were identified including 50 synonymous, 76 nonsynonymous, one stop gain, one frameshift, four splicing, 128 3’UTR, and 51 5’UTR variants. Of these, 84 rare protein-altering variants that qualified for burden analysis were identified, and all genes had a least one such variant. Five genes (*AFMID*, *HAAO*, *KYAT1/CCBL1*, *TPH1* and *WARS*) showed a burden of qualifying variants in SALS cases compared to the gnomAD nNFE dataset, however, this was not replicated when compared to the Australian control cohorts (**Table 1**). Nine novel variants in six genes were identified, each in a single individual (**Table 2**). *In silico* assessment of novel missense variants indicated that three variants present in *GOT2* (1), *KYNU* (1) and *MAOA* (1) were predicted to be pathogenic by more than 80% of the total protein prediction tools that provided prediction results (**Table 2**). Meta-analysis prediction scores from REVEL and BayeDel also correlated with these predictions (**Table 2**). The MAOA (X chromosome) variant was present in the heterozygous state in one female. None of these variants were present in additional ALS cohorts (*MAOA* data not present in Project MinE), nor were they previously implicated in other diseases (NCBI ClinVar database, https://www.ncbi.nlm.nih.gov/clinvar/). The novel *HAAO* intronic splicing variants were also predicted to affect splicing by altering intronic acceptor sites using Human Splicing Finder (36), and to be deleterious by MutationTaster, CADD and BayesDel.

**Table 1:** Burden of qualifying variants in sporadic ALS compared to controls.

Gene	SALS variants (%)	nNFE variants (%)	nNFE p-value	AOGC variants (%)	AOGC p-value	MGRB variants (%)	MGRB p-value
*AFMID*	8 (1.30)	155 (0.30)	0.0023	9 (0.93)	0.6181	11 (0.96)	0.6294
*HAAO*	7 (1.14)	97 (0.19)	0.0012	3 (0.31)	0.0546	18 (1.57)	0.5328
*KYAT1/CCBL1*	11 (1.79)	173 (0.34)	0.0001	8 (0.83)	0.1010	15 (1.31)	0.4166
*TPH1*	8 (1.30)	121 (0.23)	0.0005	8 (0.83)	0.4414	12 (1.05)	0.6425
*WARS*	8 (1.30)	146 (0.28)	0.0022	6 (0.62)	0.1773	19 (1.66)	0.6858
*AADAT*	1 (0.16)	80 (0.16)	1	2 (0.21)	1	3 (0.26)	1
*ACMSD*	2 (0.33)	110 (0.21)	0.6744	1 (0.10)	0.5639	4 (0.35)	1
*DDC*	4 (0.65)	171 (0.33)	0.3192	5 (0.52)	0.7425	6 (0.52)	0.7469
*GOT2*	1 (0.16)	111 (0.22)	1	4 (0.41)	0.6544	4 (0.35)	0.6635
*IDO1/INDO*	6 (0.98)	152 (0.29)	0.0338	4 (0.41)	0.2012	4 (0.35)	0.1083
*IDO2/INDOL1*	3 (0.49)	129 (0.25)	0.4422	7 (0.72)	0.7492	8 (0.70)	0.7567
*KMO*	7 (1.14)	126 (0.24)	0.0030	6 (0.62)	0.2708	10 (0.87)	0.6141
*KYAT3/CCBL2*	5 (0.81)	129 (0.25)	0.0457	2 (0.21)	0.1176	11 (0.96)	1
*KYNU*	2 (0.33)	170 (0.33)	1.0000	7 (0.72)	0.4958	7 (0.61)	0.5088
*MAOA*	3 (0.49)	60 (0.12)	0.1143	3 (0.31)	0.6830	1 (0.09)	0.1264
*QPRT*	2 (0.33)	90 (0.17)	0.3725	n/a*	n/a	17 (1.49)	0.0279
*TDO2*	4 (0.65)	138 (0.27)	0.1471	4 (0.41)	0.7186	12 (1.05)	0.5991
*TPH2*	2 (0.33)	134 (0.26)	0.7143	6 (0.62)	0.4958	1 (0.09)	0.2814

*Insufficient data available to calculate gene burden.

**Table 2:** Novel protein-altering variants in sporadic ALS cases.

Gene	hg19 physical position	Type	Accession number	cDNA change	protein change	Score (# tools with results)	REVEL prediction	BayesDel prediction
GOT2	chr16:58756092	exonic	NM_002080	c.G337A	p.A113T	0.92 (12)	Pathogenic	Damaging
HAAO	chr2:43010561	splicing	NM_012205	c.244-1G>C	.	1 (4)	n/a	Damaging
HAAO	chr2:43011008	splicing	NM_012205	c.160-1G>C	.	1 (4)	n/a	Damaging
KYNU	chr2:143799665	exonic	NM_003937	c.A1322G	p.Y441C	0.83 (12)	Pathogenic	Damaging
MAOA	chrX:43571952	exonic	NM_000240	c.A412T	p.I138F	0.83 (12)	Pathogenic	Damaging
TPH2	chr12:72332852	exonic	NM_173353	c.A86G	p.Q29R	0.58 (12)	Benign	Tolerated
WARS	chr14:100835432	exonic	NM_004184	c.G91A	p.A31T	0.5 (12)	Benign	Tolerated
WARS	chr14:100828251	exonic	NM_004184	c.T107C	p.I36T	0.33 (12)	Benign	Tolerated
WARS	chr14:100801280	exonic	NM_004184	c.A1348C	p.K450Q	0.25 (12)	Benign	Tolerated

n/a, not available

## Discussion

We sought to determine the prevalence of novel genetic variants or burden of rare protein-altering variants in genes that play a key role in TRP metabolism or the KP in Australian sporadic ALS. Nine novel genetic variants (absent from public control databases, including population-specific controls) were identified in *WARS* (protein synthesis)*, TPH2* and *MAOA* (serotonin synthesis), and *GOT2*, *KYNU* and *HAAO* (KP) (**Table 2**). The genes *WARS* and *TPH1*, and KP genes *AFMID, HAAO*, and *KYAT1/CCBL1* were shown to have a significant burden of qualifying rare protein-altering variants in sporadic ALS compared to the non-neuronal Non-Finnish European subset of the gnomAD dataset (**Table 1**), although this was not replicated when compared to Australian controls. This may be due to technical differences in data generation (whole-exome, PCR-amplified or PCR-free whole-genome sequencing), sample size or unidentified differences in population structure due to the highly multicultural and diverse Australian population. The increased burden of rare protein-altering variants, including the presence of novel variants, provides support for the role of TRP metabolism and the KP in ALS, and suggests these variants may act to increase risk of developing disease.

Aminoacyl-tRNA synthetases (ARSs) such as WARS are responsible for the first step of translation and protein synthesis. Mutations in the tryptophan ARS gene, *WARS*, have been found to cause the neurodegenerative disease, distal hereditary motor neuropathy (17, 18). *WARS* mutations were found to negatively affect protein synthesis and cell viability and cause neurite degeneration in neuronal cell lines and rat motor neurons (17, 18). We identified three additional novel *WARS* variants in sporadic ALS cases. Two variants (c.G91A, p.A31T and c.T107C, p.I36T) were located in close proximity within the N-terminal helix-turn-helix (WHEP) domain, responsible for protein-protein interactions (17). Interestingly, deletion of the WHEP domain of a *Caenorhabditis elegans* glycyl-tRNA synthetase was found to affect protein structure and reduce enzyme function (38). However, these *WARS* WHEP domain variants were predicted to benign by protein prediction software tools, and therefore, further analysis is required to establish their potential pathogenicity.

The neurotransmitter serotonin acts as a critical mood regulator, with its depletion highly associated with depression. This depletion may be a result of decreased availability of TRP due to activation of IDO1 and the KP, which is associated with neuroinflammation and psychological or physiological (illness) stress (39, 40). Four enzymes are involved in serotonin synthesis from TRP, with TPH1/TPH2 converting TRP to serotonin precursor 5-hydroxytrypophan (5-HTP), and MAOA converting 5-HTP to 5-hydroxyindoleacetic acid (5-HIAA, **Figure 1**). Serotonin depletion has also been associated with neurodegenerative diseases including Alzheimer’s disease and frontotemporal dementia. Decreased levels of serotonin and 5-HIAA have also been found in the spinal cord of ALS patients (41, 42), as well as in ALS patient platelets, with serotonin levels positively correlating with improved survival (41). Interestingly, administration of 5-HTP in an ALS *SOD1* mouse model significantly improved phenotype, which also corresponded with increased platelet serotonin levels in the animals (43). In an alternate ALS *SOD1* mouse model, degeneration of serotonergic neurons in the brainstem was found to lead to spasticity, a common clinical feature of ALS. Expression of mutant *SOD1* caused a loss of serotonergic neurons in the brainstem, a phenotype that was rescued with *SOD1* deletion. This, in turn, abolished spasticity in the mouse (44). We found a burden of qualifying variants in *TPH1*, and novel variants in *TPH2* and *MAOA* in sporadic ALS cases compared to controls. These genes encode tryptophan hydroxylases (*TPH*s) involved in 5-HTP synthesis and 5-HIAA synthesis respectively. Additionally, the *MAOA* variant, p.I138F was predicted to have a pathogenic effect by eight prediction tools (**Table 2**).

In the central nervous system, neuroinflammatory conditions result in increased numbers of M1 neurotoxic microglia, which produce excessive levels of QUIN (45). QUIN acts to agonise the N-methyl-D-aspartate (NMDA) receptor, resulting in an excitotoxic cascade that ultimately results in neuronal death (45). Mechanisms of QUIN neurotoxicity include protein dysfunction, oxidative stress, glutamate excitotoxicity, mitochondrial dysfunction, neuroinflammation, autophagy and apoptosis (46, 47). In ALS, several studies have found increased levels of QUIN in the CSF of patients as well as in spinal cord neuronal and microglial cells (46). Additionally, increased levels of QUIN by intracerebral injection into rat striatum resulted in increased astrocyte expression of the major ALS protein, SOD1. As a free superoxide radical scavenger, the increased SOD1 levels were thought to be a neuroprotective response to limit QUIN oxidative toxicity, a function that may be inhibited by ALS-causing mutant SOD1 protein forms (46, 48). QUIN excitotoxicity can partly be mediated by KYNA, which is produced by astrocytes (49). Interestingly, KYNA levels were also found to be higher in ALS patient CSF compared to controls, which may reflect an astroglial attempt to produce the neuroprotective metabolite (13). In serum, however, KYNA levels were conversely found to be significantly lower in ALS patients with severe clinical status compared to both patients with mild clinical status and controls (23). In a separate study, we have found similarly decreased levels of KYNA in the serum of patients with ALS as compared to controls (n= 238, p <0.001, Student’s T-test; data not shown). Of the five KP genes found to carry novel variants and/or a significant burden of qualifying variants in this study, four were directly involved in KYNA (*KYAT1/CCBL1* and *GOT2*) and QUIN (*KYNU* and *HAAO*) synthesis from 3-hydroxykynurenine (**Figure 1**).

The role of TRP and the KP in neuroinflammation, and its link to several major neurodegenerative diseases including ALS has been widely studied. We have shown for the first time that genetic variation in these genes may be associated with sporadic ALS and may confer risk to developing disease, however replication in additional cohorts is required to confirm this relationship. The protein-altering variants in the genes involved in these pathways may trigger functional effects that influence disease risk and when combined with other pathogenic ‘steps’ may progressively lead to ALS onset. Further studies can now commence to determine the specific pathogenic role of the novel variants and genes that carry a burden of variants in sporadic ALS.

## Data Availability Statement 

The original contributions presented in the study are included in the article/supplementary material. Further inquiries can be directed to the corresponding author.

## Ethics Statement

The studies involving human participants were reviewed and approved by human research ethics committees of Macquarie University (5201600387), Sydney South West Area Health District and The University of Sydney. The patients/participants provided their written informed consent to participate in this study.

## Author Contributions

JF, IF and GG conceptualised and designed the studies and experiments. Experiments were performed by JF, SC, and EM. Data was curated by KW, NT, DB and EM. Data was analysed by JF, SC and VT. Resources were obtained by RP, MK and DR. JF wrote the manuscript. All authors contributed to the article and approved the submitted version. IB supervised the project and acquired funding.

## Funding

This work was funded by Motor Neuron Disease Research Australia (Bill Gole Postdoctoral Research Fellowship), the National Health and Medical Research Council of Australia (1092023, 1156093, 1176913, 1132524, 1153439, 1095215, 1176660) and Macquarie University.

## Conflict of Interest

The authors declare that the research was conducted in the absence of any commercial or financial relationships that could be construed as a potential conflict of interest.

## References

[1] Shefner JM Al-Chalabi A Baker MR Cui L-Y de Carvalho M Eisen A. A Proposal for New Diagnostic Criteria for ALS. Clin Neurophysiol (2020) 131:1975–8. 10.1016/j.clinph.2020.04.005 32387049

[2] van Es MA Hardiman O Chio A Al-Chalabi A Pasterkamp RJ Veldink JH. Amyotrophic Lateral Sclerosis. Lancet (2017) 390:2084–98. 10.1016/S0140-6736(17)31287-4 28552366

[3] Gregory JM Fagegaltier D Phatnani H Harms MB. Genetics of Amyotrophic Lateral Sclerosis. Curr Genet Med Rep (2020) 8:121–31. 10.1007/s40142-020-00194-8

[4] McCann EP Henden L Fifita JA Zhang KY Grima N Bauer DC. Evidence for Polygenic and Oligogenic Basis of Australian Sporadic Amyotrophic Lateral Sclerosis. J Med Genet (2021) 58:87–95. 10.1136/jmedgenet-2020-106866 32409511

[5] McCann EP Williams KL Fifita JA Tarr IS O’Connor J Rowe DB. The Genotype-Phenotype Landscape of Familial Amyotrophic Lateral Sclerosis in Australia. Clin Genet (2017) 92:259–66. 10.1111/cge.12973 28105640

[6] Mathis S Goizet C Soulages A Vallat J-M Masson GL. Genetics of Amyotrophic Lateral Sclerosis: A Review. J Neurol Sci (2019) 399:217–26. 10.1016/j.jns.2019.02.030 30870681

[7] Al-Chalabi A Fang F Hanby MF Leigh PN Shaw CE Ye W. An Estimate of Amyotrophic Lateral Sclerosis Heritability Using Twin Data. J Neurol Neurosurg Psychiatry (2010) 81:1324–6. 10.1136/jnnp.2010.207464 PMC2988617 20861059

[8] Wingo TS Cutler DJ Yarab N Kelly CM Glass JD. The Heritability of Amyotrophic Lateral Sclerosis in a Clinically Ascertained United States Research Registry. PloS One (2011) 6(11):e27985. 10.1371/journal.pone.0027985 e.27985. 22132186 PMC3222666

[9] Ryan M Heverin M McLaughlin RL Hardiman O. Lifetime Risk and Heritability of Amyotrophic Lateral Sclerosis. JAMA Neurol (2019) 76:1367–74. 10.1001/jamaneurol.2019.2044 PMC6646974 31329211

[10] Al-Chalabi A Calvo A Chio A Colville S Ellis CM Hardiman O. Analysis of Amyotrophic Lateral Sclerosis as a Multistep Process: A Population-Based Modelling Study. Lancet Neurol (2014) 13:1108–13. 10.1016/S1474-4422(14)70219-4 PMC4197338 25300936

[11] Chiò A Mazzini L D’Alfonso S Corrado L Canosa A Moglia C. The Multistep Hypothesis of ALS Revisited. Neurology (2018) 91:e635–42. 10.1212/WNL.0000000000005996 PMC6105040 30045958

[12] Cirulli ET Lasseigne BN Petrovski S Sapp PC Dion PA Leblond CS. Exome Sequencing in Amyotrophic Lateral Sclerosis Identifies Risk Genes and Pathways. Science (2015) 347(6229):1436–41. 10.1126/science.aaa3650 PMC4437632 25700176

[13] Guillemin GJ Meininger V Brew BJ. Implications for the Kynurenine Pathway and Quinolinic Acid in Amyotrophic Lateral Sclerosis. Neurodegener Dis (2006) 2:166–76. 10.1159/000089622 16909022

[14] Chen Y Stankovic R Cullen KM Meininger V Garner B Coggan S. The Kynurenine Pathway and Inflammation in Amyotrophic Lateral Sclerosis. Neurotox Res (2010) 18(2):132–42. 10.1007/s12640-009-9129-7 19921535

[15] Kalisch BE Jhamandas K Boegman RJ Beninger RJ. Picolinic Acid Protects Against Quinolinic Acid-Induced Depletion of NADPH Diaphorase Containing Neurons in the Rat Striatum. Brain Res (1994) 668:1–8. 10.1016/0006-8993(94)90504-5 7535651

[16] Maddison DC Giorgini F. The Kynurenine Pathway and Neurodegenerative Disease. Semin Cell Dev Biol (2015) 40:134–41. 10.1016/j.semcdb.2015.03.002 25773161

[17] Tsai PC Soong BW Mademan I Huang YH Liu CR Hsiao CT. A Recurrent WARS Mutation is a Novel Cause of Autosomal Dominant Distal Hereditary Motor Neuropathy. Brain (2017) 140:1252–66. 10.1093/brain/awx058 PMC6248622 28369220

[18] Wang B Li X Huang S Zhao H Liu J Hu Z. A Novel WARS Mutation (p.Asp314Gly) Identified in a Chinese Distal Hereditary Motor Neuropathy Family. Clin Genet (2019) 96:176–82. 10.1111/cge.13563 31069783

[19] Boros FA Bohár Z Vécsei L. Genetic Alterations Affecting the Genes Encoding the Enzymes of the Kynurenine Pathway and Their Association With Human Diseases. Mutat Res (2018) 776:32–45. 10.1016/j.mrrev.2018.03.001 29807576

[20] Lim CK Bilgin A Lovejoy DB Tan V Bustamante S Taylor BV. Kynurenine Pathway Metabolomics Predicts and Provides Mechanistic Insight Into Multiple Sclerosis Progression. Sci Rep (2017) 7:41473. 10.1038/srep41473 28155867 PMC5290739

[21] Lim CK Fernández-Gomez FJ Braidy N Estrada C Costa C Costa S. Involvement of the Kynurenine Pathway in the Pathogenesis of Parkinson’s Disease. Prog Neurobiol (2017) 155:76–95. 10.1016/j.pneurobio.2015.12.009 27072742

[22] Guillemin GJ Brew BJ Noonan CE Takikawa O Cullen KM. Indoleamine 2,3 Dioxygenase and Quinolinic Acid Immunoreactivity in Alzheimer’s Disease Hippocampus. Neuropathol Appl Neurobiol (2005) 31:395–404. 10.1111/j.1365-2990.2005.00655.x 16008823

[23] Iłzecka J Kocki T Stelmasiak Z Turski WA. Endogenous Protectant Kynurenic Acid in Amyotrophic Lateral Sclerosis. Acta Neurol Scand (2003) 107:412–8. 10.1034/j.1600-0404.2003.00076.x 12757473

[24] Tan VX Guillemin GJ. Kynurenine Pathway Metabolites as Biomarkers for Amyotrophic Lateral Sclerosis. Front Neurosci (2019) 10:1013. 10.3389/fnins.2019.01013 PMC6764462 31616242

[25] Brooks BR Miller RG Swash M Munsat TL. El Escorial Revisited: Revised Criteria for the Diagnosis of Amyotrophic Lateral Sclerosis. Amyotroph Lateral Scler (2000) 1:293–9. 10.1080/146608200300079536 11464847

[26] Lek M Karczewski KJ Minikel EV Samocha KE Banks E Fennell T. Analysis of Protein-Coding Genetic Variation in 60,706 Humans. Nature (2016) 536:285–91. 10.1038/nature19057 PMC5018207 27535533

[27] Pinese M Lacaze P Rath EM Stone A Brion M-J Ameur A. The Medical Genome Reference Bank Contains Whole Genome and Phenotype Data of 2570 Healthy Elderly. Nat Commun (2020) 11:435. 10.1038/s41467-019-14079-0 31974348 PMC6978518

[28] Wang K Li M Hakonarson H. Annovar: Functional Annotation of Genetic Variants From High-Throughput Sequencing Data. Nucleic Acids Res (2010) 38:e164. 10.1093/nar/gkq603 20601685 PMC2938201

[29] Liu X Wu C Li C Boerwinkle E. dbNSFP V3.0: A One-Stop Database of Functional Predictions and Annotations for Human Nonsynonymous and Splice-Site Snvs. Hum Mutat (2016) 37:235–41. 10.1002/humu.22932 PMC4752381 26555599

[30] Trost B Walker S Wang Z Thiruvahindrapuram B MacDonald JR Sung WWL. A Comprehensive Workflow for Read Depth-Based Identification of Copy-Number Variation From Whole-Genome Sequence Data. Am J Hum Genet (2018) 102:142–55. 10.1016/j.ajhg.2017.12.007 PMC5777982 29304372

[31] RStudio Team. Rstudio: Integrated Development for R (2010). Available at: http://www.rstudio.com.

[32] Robinson JT Thorvaldsdóttir H Winckler W Guttman M Lander ES Getz G. Integrative Genomics Viewer. Nat Biotechnol (2011) 29:24–6. 10.1038/nbt.1754 PMC3346182 21221095

[33] Ioannidis NM Rothstein JH Pejaver V Middha S McDonnell SK Baheti S. Revel: An Ensemble Method for Predicting the Pathogenicity of Rare Missense Variants. Am J Hum Genet (2016) 99:877–85. 10.1016/j.ajhg.2016.08.016 PMC5065685 27666373

[34] Feng B-J. Perch: A Unified Framework for Disease Gene Prioritization. Hum Mutat (2017) 38:243–51. 10.1002/humu.23158 PMC5299048 27995669

[35] Tian Y Pesaran T Chamberlin A Fenwick RB Li S Gau C-L. REVEL and BayesDel Outperform Other *In Silico* Meta-Predictors for Clinical Variant Classification. Sci Rep (2019) 9:12752. 10.1038/s41598-019-49224-8 31484976 PMC6726608

[36] Desmet F-O Hamroun D Lalande M Collod-Béroud G Claustres M Béroud C. Human Splicing Finder: An Online Bioinformatics Tool to Predict Splicing Signals. Nucleic Acids Res (2009) 37:e67. 10.1093/nar/gkp215 19339519 PMC2685110

[37] Van Rheenen W Pulit SL Dekker AM Al Khleifat A Brands WJ Iacoangeli A. Project MinE: Study Design and Pilot Analyses of a Large-Scale Whole-Genome Sequencing Study in Amyotrophic Lateral Sclerosis. Eur J Hum Genet (2018) 26:1537–46. 10.1038/s41431-018-0177-4 PMC6138692 29955173

[38] Chang CY Chien CI Chang CP Lin BC Wang CC. A WHEP Domain Regulates the Dynamic Structure and Activity of *Caenorhabditis elegans* glycyl-tRNA Synthetase. J Biol Chem (2016) 291:16567–75. 10.1074/jbc.M116.730812 PMC4974372 27298321

[39] Lovelace MD Varney B Sundaram G Lennon MJ Lim CK Jacobs K. Recent Evidence for an Expanded Role of the Kynurenine Pathway of Tryptophan Metabolism in Neurological Diseases. Neuropharmacology (2017) 112:373–88. 10.1016/j.neuropharm.2016.03.024 26995730

[40] Myint AM Kim YK. Cytokine-Serotonin Interaction Through IDO: A Neurodegeneration Hypothesis of Depression. Med Hypotheses (2003) 61:519–25. 10.1016/S0306-9877(03)00207-X 14592780

[41] Dupuis L Spreux-Varoquaux O Bensimon G Jullien P Lacomblez L Salachas F. Platelet Serotonin Level Predicts Survival in Amyotrophic Lateral Sclerosis. PloS One (2010) 5:e13346. 10.1371/journal.pone.0013346 20967129 PMC2954194

[42] Sandyk R. Serotonergic Mechanisms in Amyotrophic Lateral Sclerosis. Int J Neurosci (2006) 116:775–826. 10.1080/00207450600754087 16861147

[43] Turner BJ Lopes EC Cheema SS. The Serotonin Precursor 5-Hydroxytryptophan Delays Neuromuscular Disease in Murine Familial Amyotrophic Lateral Sclerosis. Amyotroph Lateral Scler Other Mot Neuron Disord (2003) 4:171–76. 10.1080/14660820310009389 14527871

[44] El Oussini H Scekic-Zahirovic J Vercruysse P Marques C Dirrig-Grosch S Dieterlé S. Degeneration of Serotonin Neurons Triggers Spasticity in Amyotrophic Lateral Sclerosis. Ann Neurol (2017) 82:444–56. 10.1002/ana.25030 28856708

[45] Jacobs KR Lovejoy DB. Inhibiting the Kynurenine Pathway in Spinal Cord Injury: Multiple Therapeutic Potentials? Neural Regener Res (2018) 13:2073–6. 10.4103/1673-5374.241446 PMC6199950 30323124

[46] Lee JM Tan V Lovejoy D Braidy N Rowe DB Brew BJ. Involvement of Quinolinic Acid in the Neuropathogenesis of Amyotrophic Lateral Sclerosis. Neuropharmacology (2017) 112:346–64. 10.1016/j.neuropharm.2016.05.011 27265569

[47] Guillemin GJ. Quinolinic Acid, the Inescapable Neurotoxin. FEBS J (2012) 279:1356–65. 10.1111/j.1742-4658.2012.08485.x 22248144

[48] Bruijn LI Miller TM Cleveland DW. Unraveling the Mechanisms Involved in Motor Neuron Degeneration in ALS. Annu Rev Neurosci (2004) 27:723–49. 10.1146/annurev.neuro.27.070203.144244 15217349

[49] Guillemin GJ Kerr SJ Smythe GA Smith DG Kapoor V Armati PJ. Kynurenine Pathway Metabolism in Human Astrocytes: A Paradox for Neuronal Protection. J Neurochem (2001) 78:842–53. 10.1046/j.1471-4159.2001.00498.x 11520905

